# Imaging features of the renal lymphoma: case report and literature review

**DOI:** 10.1259/bjrcr.20220117

**Published:** 2023-04-04

**Authors:** Shaden Saleh Almousa, Ammar Ashraf, Ahmed Mohamed Abdelrahman, Mohamed Tahar Yacoubi

**Affiliations:** 1 Department of Medical Imaging, King Faisal University, Al-Ahsa, KSA, Saudi Arabia; 2 Department of Medical Imaging, King Abdulaziz National Guard Hospital, Al-Ahsa, KSA, Saudi Arabia; 3 Department of Anatomical Pathology, King Abdulaziz National Guard Hospital, Al-Ahsa, KSA, Saudi Arabia

## Abstract

Renal affection is common in disseminated non-Hodgkin’s lymphoma (NHL) which is known as secondary renal lymphoma (SRL). Primary renal lymphoma (PRL) is an exceedingly uncommon disease, which accounts for less than 1% of all renal masses. Diffuse large B-cell lymphoma (DLBCL) is the most common subtype of NHL in both primary as well as secondary renal lymphomas. PRL is of paramount importance clinically as it is usually managed with neo-adjuvant chemotherapy followed by nephrectomy in contrast to the more frequently seen renal cell carcinoma, which is treated surgically. This outstanding difference in management challenges the longstanding approach that preoperative biopsies are not mandatory prior to nephrectomy for renal masses.

Because of its relative rarity, the imaging features of PRL have been described in a few studies, and having an understanding of these typical imaging patterns is crucial for making an accurate diagnosis and differentiation from other renal malignancies.

Here, we present a case of a secondary renal lymphoma and discuss its differential imaging features.

## Introduction

Renal involvement is commonly seen in the known cases of lymphoma, particularly those with disseminated nodal or extranodal disease. This involvement is known as secondary renal lymphoma (SRL), and is usually caused by direct invasion or hematogenous spread.^1,2^ The incidence of secondary renal lymphoma (SRL) is approximately 3% and is seen more frequently in patients with non-Hodgkin lymphoma (NHL), with the predominant subtype being diffuse large B-cell lymphoma (DLBCL).^1,2^ Rarely, lymphoma may involve only the kidneys and shows no evidence of extrarenal visceral or nodal disease; such renal involvement is known as primary renal lymphoma (PRL).^1–5^ Primary renal lymphoma (PRL) is an exceedingly uncommon disease that accounts for less than 1% of all cases of extranodal lymphoma.^1–9^

To date, approximately 100 cases of primary renal lymphoma (PRL), including both Hodgkin’s disease and non-Hodgkin’s lymphoma, have been published, and most are diffuse large B-cell lymphomas (DLBCLs).^3,5–7^

Diagnosis of PRL can be quite laborious; however, familiarity with its spectrum of radiological features, can help distinguish PRL from the other common primary renal malignant lesions, such as renal cell carcinoma (RCC).^8^ This distinction between PRL and RCC is crucial because of the striking difference in their management, *i.e*. renal lymphoma is primarily treated with chemotherapy, whereas the RCC is usually managed with surgery or radiofrequency ablation (RFA), depending on the stage of the disease.^1,2,4,5,8^

## Case presentation

**Figure 1.**
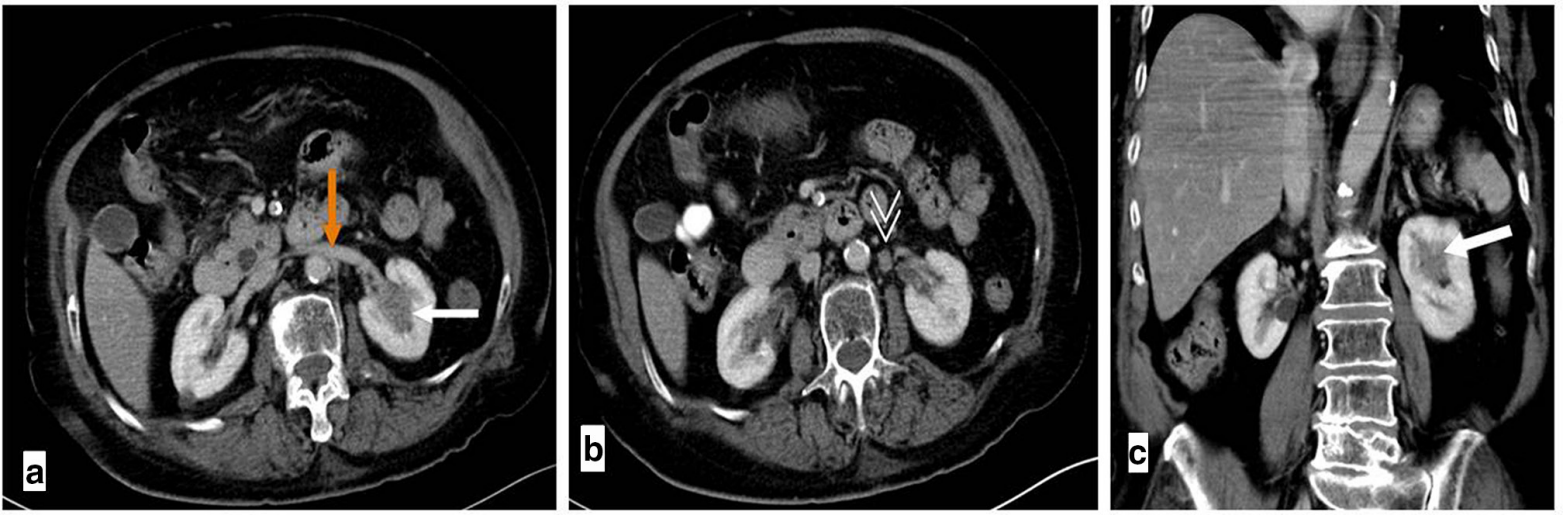
Contrast-enhanced axial (**a, b**) and coronal (**c**) CT scan images showing a small ill-defined non-enhancing mass in the left renal sinus (white arrows). Small loco-regional lymph node (white arrowhead in B) adjacent to the patent left renal vein (orange arrow in A). Abbreviations: CT, computed tomography.

A 90-year-old female presented to our hospital with a short history of abdominal pain and bloody diarrhea. During investigations of these symptoms, a small ill-defined non-enhancing mass, suspicious of renal cell carcinoma, was discovered incidentally, in the left renal sinus on the CT of the abdomen (Figure 1). This renal lesion was associated with a few sub centimeter lymph nodes along the patent left renal vessels. The patient failed to follow up, and 9 months later, she presented to our hospital’s emergency department with a complaint of moderate intensity intermittent left flank pain, which was getting worse over the last three months. The pain was associated with nausea. There was also history of hematuria, fever, malaise, and approximately 10 kg of weight loss in the last three months. Her past medical history was positive for peptic ulcer disease, benign biliary stricture, and recurrent urinary tract infections. Urinalysis revealed urinary tract infection and hematuria, and the urine culture showed heavy growth of Escherichia coli. Other positive findings in the laboratory investigations were: C-reactive protein (CRP) = 204 mg l^−1^ (normal range≤1.20), serum albumin = 18 g l^−1^ (normal range34‒48), total protein = 49 g l^−1^ (normal range 60‒83), hemoglobin = 72 g l^−1^ (normal range 120‒160), lactic acid = 1.98 mmol l^−1^ (normal range 0.50‒2.20), and sodium = 133 mmol l^−1^ (normal range 136‒145). Renal parameters were within the normal limits.

**Figure 2.**
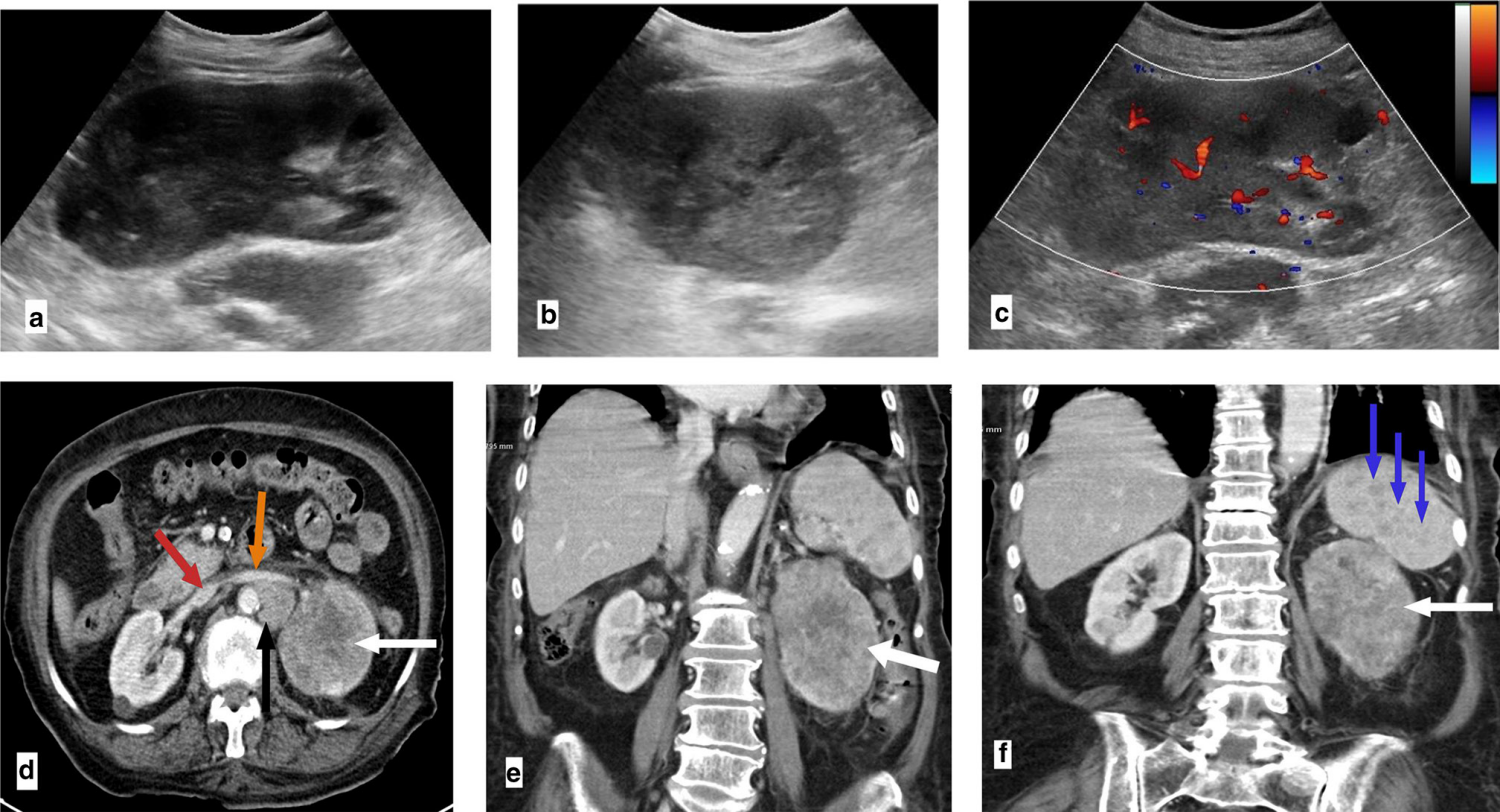
Longitudinal (**a**) and transverse (**b**) grey scale US images showing a poorly defined infiltrating heterogeneous hypoechoic lesion at the upper and mid poles of the left kidney. Colour Doppler US examination (**c**) showed intralesional vascularity. The normal shape of the kidney was preserved. Axial (**d**) and coronal portovenous (**e, f**) Phase CT images showing the relatively enlarged left kidney with an infiltrating poorly enhancing mass lesion that almost completely replaces its upper and mid poles (white arrows). Patent left renal vein (orange arrow in D) and IVC (red arrow in D). Enlarged lymph node (black arrow in D) adjacent to the left renal vein. Multiple small focal hypodense lesions in the spleen (blue arrows in F). Abbreviation: US, ultrasound; IVC, inferior vena cava.

Renal ultrasound examination was performed which showed a relatively bulky left kidney with a poorly circumscribed heterogeneous hypoechoic mass lesion at its upper and mid poles. The lesion had mild internal vascularity upon colour Doppler ultrasound examination (Figure 2). No renal calculi or hydronephrosis was observed.

**Figure 3.**
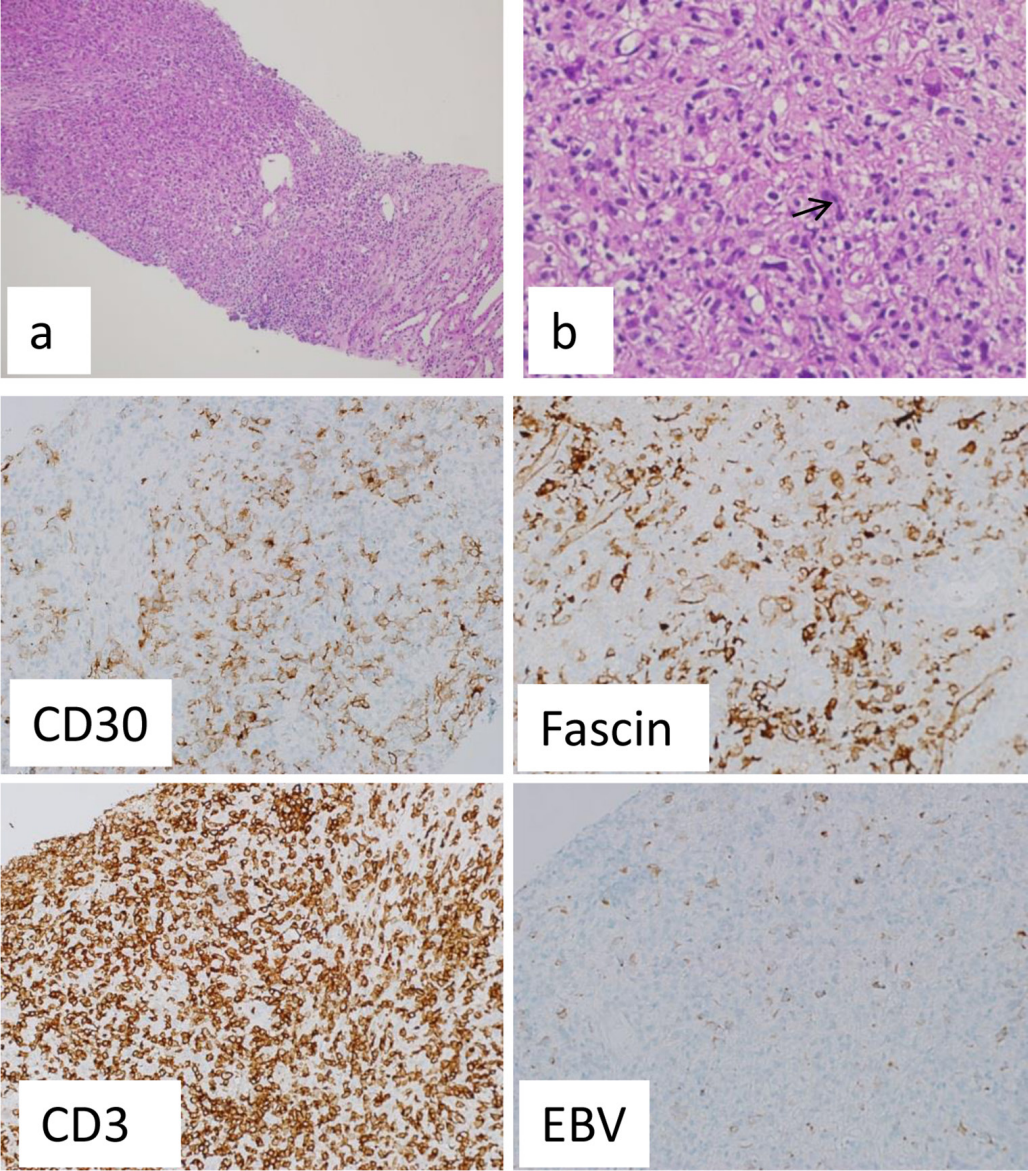
H & E staining x 100 (**a**) and H & E staining x 400 (**b**). Infiltration of kidney by scattered large malignant cells having Reed-Sternberg features (→ in b). The background shows polymorphous inflammatory cells. These large cells express CD30 and FASCIN. The background is rich with T-cells, expressing CD3. Many large cells are EBV+.(Figure 3)

A contrast-enhanced CT scan of the abdomen was performed, which revealed a dramatic increase in the size of the left renal mass and lymphadenopathy along the left renal vessels. The renal mass was poorly enhancing and was almost completely replacing the upper and mid poles of the left kidney, due to its infiltrative nature. There was also interval development of multiple small focal lesions within the average size spleen. Based on these imaging features (large non-calcified poorly enhancing renal mass with lymphadenopathy along the renal vessels, patent renal vessels, and multiple splenic lesions), suspicion of renal lymphoma was raised, and imaging guided biopsy was advised. Ultrasound-guided biopsy of the left renal mass was performed, and histopathology revealed multiple Reed-Sternberg and Hodgkin cells. Multiple areas of necrosis were also noted. Immunohistochemical stains revealed that the neoplastic cells were immunoreactive to Vimentin, CD15, and CD30. Numerous CD3 positive cells and a few scattered CD79a positive cells were also noted. The neoplastic cells were negative for cytokeratin AE1/AE3. EBV immunohistochemistry showed immunoreactivity in multiple atypical cells. Immunoreactivity for cytomegalovirus was negative. Based on the morphology and immunohistochemical pattern, a diagnosis of Hodgkin-type renal lymphoma was made. No hepatosplenomegaly or significant lymphadenopathy was observed in the lower chest and the remaining abdominopelvic cavity. The family refused bone marrow biopsy because of the old age of the patient. The patient was referred to the medical oncologist; however, she died three weeks later without any further work-up or treatment.

## Discussion

In disseminated lymphoma, after the hematopoietic and reticuloendothelial system, the genitourinary system is the second most frequently affected anatomical system, and within the genitourinary system, the kidney is the most common affected organ/site.^1,2,4,8,9^ Any subtype of the lymphoma can infiltrate the kidneys; however, non-Hodgkin lymphoma, usually of the DLBCL subtype, is the most common secondary and primary renal lymphoma.^1–5,7–9^ The marginal zone lymphoma (MZL) is the second most common histopathological subtype of PRL.^6^ Renal involvement is quite uncommon in a Hodgkin lymphoma.^1,2,4,8,9^ In contrast to PRL, SRL is commonly observed in patients with disseminated disease. The reported incidence of SRL is 30–60% in autopsy series; however, such renal involvement has imaging documentation in only 3–8% of cases.^1,2,4–6,8,9^ This broad discrepancy between the autopsy and radiology literature is multifactorial and may be explained by clinically silent renal disease, suboptimal imaging detection of renal disease by routine anatomical imaging, and lack of tissue diagnosis in the background of disseminated disease.^1,2,4,5,8,9^ Immunosuppression (organ transplantation) and an immunocompromised status (HIV infection) are possible predisposing factors of renal lymphoma.^1,2,4,8^ To avoid confusion between SRL and PRL, a diagnostic criteria has been designed for PRL, which includes: renal infiltration by the lymphomatous process, non-obstructive enlargement of one or both kidneys, and absence of extrarenal visceral or nodal disease.^1,2,4–6,8,9^ The gold standard for diagnosis of renal lymphoma is renal biopsy.^3^ According to this diagnostic criteria and last CT scan findings, our case is secondary renal lymphoma.

The exact aetiology of PRL is controversial, as the kidney is an extranodal viscera and is devoid of lymphoid tissue typically.^1–9^ It is hypothesized that PRL likely arises from a lymphoid-rich capsule of the kidney or fat around the kidney and subsequently invades the renal parenchyma. The lymphocytes observed in the region of chronic renal inflammation have also been proposed as a potential causative factor, and this process resembles extranodal mucosa-associated lymphoid tissue (MALT) lymphomas, affecting the non-lymphoid tissues, such as skin and breast.^1–5,7–9^

PRL more frequently affects middle-aged people, with the age of presentation usually being above 40 years; however, there are case reports of PRL in patients as young as 21 years old.^1,2,4–8^ Immunocompromised patients usually present at younger age.^2^ PRL is more common in males with a male: female ratio of ~1.6:1 to 2:1.^1,2,4–8^

PRL is often asymptomatic and shows normal renal functions.^1,2,4,8^ However, it can present with flank/abdominal pain, palpable abdominal mass, gross hematuria, proteinuria, renal failure, hypertension, night sweats, fatigue, fever, malaise, or weight loss, with the most common symptoms being pain and fever.^1–9^ In adult patients (18–50 years), abdominal/flank pain (62%) is the most common symptom, in younger patients (<18 years), fever (56%) is the most common symptom, and in the older patients (>50 years), weight loss and gross hematuria (37%) are the most common symptoms.^5^ There are no specific clinical features of PRL, and sometimes, it can mimic renal abscess, renal cell carcinoma (RCC), and renal metastases.^5^

The imaging features of primary and secondary renal lymphoma are similar except that the SRL has associated widespread extrarenal disease.^1,2,4,5,8^ An unambiguous diagnosis of PRL requires histopathological validation, bone marrow biopsy, and staging whole-body scan to exclude the presence of extrarenal disease at the time of diagnosis.^2^ Renal lymphoma has various radiological appearances, including multiple or solitary focal renal lesions, direct renal extension from the retroperitoneal lymphadenopathy, unilateral or bilateral diffuse renal infiltration, and isolated perirenal disease, and some of these radiological features are related to the histologic growth pattern.^1,2,4,5,8,9^ The radial growth pattern of the tumor is demonstrated as an expansile mass radiologically, which may be multiple (50–60% cases) or single (10–25% cases) involving unilateral or bilateral kidneys.^1,2,4,5,7–9^ The second most frequent pattern is direct renal infiltration from contiguous retroperitoneal lymphadenopathy, which is observed in 25–30% of cases.^1,2,4,5,8,9^The infiltrative tumor growth pattern results in nephromegaly without loss of the normal renal shape due to diffuse renal infiltration. This pattern is observed in 20% of cases, affects bilateral kidneys, and commonly presents as acute renal insufficiency.^1,2,4,5,7–9^ Isolated perirenal lymphoma (not invading the renal parenchyma) is a rare exceptional form (10% of cases), that is not observed in other genitourinary viscerae.^1,2,4,5,8,9^ Predominant involvement of the renal sinus, peripelvic region, and renal collecting system with minimal collecting system dilatation regarding the size of renal disease and encasement of the renal vessels are uncommon imaging appearances of renal lymphoma.^1,2,4,8,9^ Imaging appearance as a cystic lesion (before treatment) is also an uncommon finding.^1,2,4,8,9^ Focal lymphomatous renal lesions are variable in size and commonly measure 1.0–4.5 cm in size.^1,2,4,8,9^ The majority of the renal lymphomatous lesions resolve after chemotherapy; however, cystic degeneration/necrosis and calcifications may rarely evolve. Post-treatment renal atrophy has also been described.^2^ Without any history of prior treatment, calcifications are rare in renal lymphoma.^1,2,4,8,9^ Unilateral disease is more common than bilateral disease.^3,5,6^PRL commonly appears to affect one kidney in adult patients and both kidneys in younger patients (< 18 years).^3,5^

On grey scale ultrasound examination, renal lymphoma is characteristically hypoechoic.^1,2,4,5,8,9^ Occasionally, it can be anechoic and mimic a cystic renal lesion.^1,2,4,8,9^ It is a hypovascular tumor and has minimal internal vascularity upon colour Doppler ultrasound examination.^1,2,4,8,9^ Diffuse renal enlargement may also be noted.^1,2,4,8^ It is also a good imaging tool in the assessment of patients with renal failure and provides real-time imaging guidance during percutaneous renal biopsy which has a sensitivity and specificity of 90–100% in diagnosing malignancy.^1,2,4,8,9^ Ultrasound is usually the initial imaging modality; however, due to its nonspecific imaging features, further evaluation with CT or MRI is warranted.^1,2,4,8^

Contrast enhanced CT is an effective, comprehensive, and sensitive imaging modality of choice to detect, characterize, and stage the renal lymphoma.^2,5,7–9^ It is also used to define the extent of extrarenal disease, and access the treatment response and follow-up of renal lymphoma.^5,8,9^ Classically, renal lymphoma is a hypovascular tumor, which enhances less than the normal renal cortex and is best visualized during the portovenous/nephrographic phase of the contrast-enhanced CT scan.^1,2,4–6,8,9^ It is mildly hypodense on the unenhanced CT scan.^6^ Due to their infiltrative nature, renal lymphomatous tumors can grow into the perirenal space and retroperitoneum. They can encase the nearby renal blood vessels and IVC, and exert pressure effects on them; however, invasion of these vascular structures is rare (regarding the size of the renal lesion), and this peculiar imaging feature can be used as a differentiating point between the renal lymphoma and RCC (which is usually associated with the vascular invasion).^1,2,4,6,8,9^ The main differential diagnosis of PRL (hypovascular tumor) is renal cell carcinoma (RCC), and the most common subtype of the RCC is clear cell RCC, which is a hypervascular tumor.^1,2,4,8^ Both of these lesions are hypodense/hypoenhancing on a routine CT/MRI scan, which is usually acquired in the portovenous phase, and thus, they cannot be differentiated from each other.^1,2,4,8,9^ However, acquiring images in multiple phases after administration of an IV contrast would be helpful for such differentiation, as clear-cell RCC hyper-enhances during the arterial phase, and becomes hypodense in the nephrographic phase, and thus can be differentiated from renal lymphoma.^1,2,4,8^ Uncommon low-grade renal cell carcinomas such as papillary and chromophobe RCCs, demonstrate slow progressive enhancement without any intense enhancement in the arterial phase and may mimic renal lymphoma, which is hypoenhancing in all phases of the scan.^1,2,4,8^ Spontaneous hemorrhage, cystic changes/necrosis, calcifications and heterogeneous attenuation are atypical CT findings of renal lymphoma.^5,9^

Magnetic resonance (MR) imaging, with its better soft tissue contrast compared to CT, has similar accuracy in the detection of renal lymphoma.^2^ Albeit CT scan is the preferred imaging technique in the assessment of renal lymphoma, MRI may be valuable, particularly in patients with a history of renal failure or allergy to the IV contrast medium and in children and young adults to avoid the radiation hazards of a CT scan.^1,2,4,7,8^ On MRI, renal lymphoma is hypointense on T1 and isointense to hypointense to the normal renal cortex on *T*_2_-weighted images.^1,2,4,5,8,9^ This signal intensity pattern is different from other common renal pathologies, such as renal cysts, clear cell RCCs, and cystic renal tumors, which are hyperintense on *T*_2_-weighted images.^4^ Upon post-contrast study, renal lymphoma shows mild heterogeneous enhancement, which is less than the normal renal cortex.^1,2,4,8,9^ The MR imaging features of renal lymphoma on the diffusion-weighted imaging (DWI) sequence are not well recognized; however, according to the literature, it shows restricted diffusion.^1,2,4,5,8^ The reported mean apparent diffusion coefficient (ADC) value for renal lymphoma is 0.64–0.76 x 10^−3^mm^2^/s, normal renal parenchyma is 2.18–2.30 x 10^−3^mm^2^/s, clear cell RCC is 1.23–1.70 x 10^−3^mm^2^/s, chromophobe RCC is 1.14–1.41 x 10^−3^mm^2^/s, and papillary RCC is 0.88–0.90 x 10^−3^mm^2^/s.^4,5^

Positron emission tomography (PET) with 18F‒fluorodeoxyglucose (18F‒FDG) integrated with computed tomography (18F‒FDG PET/CT) provides a combination of anatomical and functional imaging information and, is the standard of care for baseline tumor staging, assessment of the treatment response, and follow up in patients with lymphoma.^1,2,4,5,8^ The role of PET/CT in renal lymphoma has not been studied on a large‒scale to date. However, it may be helpful in differentiating renal lymphoma from the RCC as the lymphoma shows more intense FDG uptake than the RCC.^1,2,4,5,8,9^ Nicolau et al^1^ reported an SUV_max_>5.98 g ml^−1^ (median = 10.99 g ml^−1^) &SUV_mean_ > 4.68 g ml^−1^ in the renal lymphomas and an SUV_max_<5.26 g ml^−1^ (median = 2.91 g ml^−1^) & an SUV_mean_ < 3.39 g ml^−1^ in the RCCs (*p* < 0.0001). Regarding the size and margins of the tumor, they did not find any statistically significant differences between the renal lymphoma & RCC.^1^ Chen X et al^5^ also described a significant difference in the mean standardized uptake value (SUV_mean_) of PRL (SUV_mean=_6.37±2.28) and clear cell renal cell carcinoma (SUV_mean=_2.58±0.62).

Albeit the clinical efficiency of 18F‒FDG PET‒CT for the baseline staging and follow‒up of the patients with lymphoma is well recognized, whole‒body diffusion weighted (DW) MR and PET‒MR imaging are the modern emerging imaging techniques.^2^ Although the combination of CT or MRI and FDG‒PET scan findings is suggestive of malignancy; however, tissue diagnosis is eventually required to clinch the histological type of the tumour.^1,2,4,8^

The main treatment of the primary renal lymphoma is chemotherapy.^1,2,4,5,8^Nephrectomy or isolated radiotherapy have occasionally been described in the treatment of PRL.^2,5^ However, the role of surgery or radiotherapy in PRL had not been investigated on a larger scale and the present knowledge is based upon the case reports, case series and literature reviews.^3,5^ Belbaraka R et al^3,5^ preferred chemotherapy to treat the PRL over the surgery whereas Chen X. et al^5^and Chen J et al^3^ reported an excellent outcome in the patients treated surgically. No improvement was seen in the survival rate with radiotherapy.^3^

Traditionally, PRL had been associated with a poor prognosis; however, early detection and appropriate treatment can lead to a favorable outcome with improved overall 5‒year survival rates.^1,2,4,8^ The 5‒year survival rate is only 40‒50%.^2,5,7^ Prognosis depends on the histological type of the lymphoma, stage of the lymphoma, management options and age of the patient. Marginal zone lymphoma (MZL) subtype has a good prognosis when compared with the T‒cell or natural killer (NK)‒cell lymphoma subtypes; MZL has a 1‒year relative survival rate of 94% and 5‒years relative survival rate of 87% whereas T‒cell or natural killer (NK)‒cell lymphoma has a 1‒year relative survival rate of 51% only.^3,6^ Chen X et al^5^ reported a mean survival time of 15.8 months in the patients treated with chemotherapy alone and a mean survival time of 49.4 months in the patients treated with a combination of chemotherapy and surgery. They reported a mean survival rate of 62.8 months, 48.2 months and 17.6 months in patients aged 18‒50 years, > 50 years and 0‒18 years respectively.^5^ They also reported a mean survival time of 21 months in the bilateral and a mean survival time of 68 months in unilateral renal lymphoma.^5^ Younger age and bilateral PRL are associated with a shorter survival time and more rapid progression of the disease.^5^ They recommended that chemotherapy should be combined with surgery, radiotherapy or any other means, particularly in the younger patients and patients with bilateral PRL.^5^The lesion size greater than 10 cm, diffuse renal infiltration and lesion involving the renal hilum were associated with a catastrophic outcome.^1,2,4,8^ The presence of renal involvement at the time of initial diagnosis is linked with a high risk of central nervous system (CNS) relapse, which is associated with a poorer prognosis.^1,2,4,8^

Improvements have been noted in the renal functions within 2‒4 weeks of chemotherapy.^1,2,4,5,7,8^

In conclusion, renal lymphoma is an uncommon diagnosis with various radiological appearances creating two main challenges for the radiologists. First challenge is the differentiation between therenal lymphoma and RCC and the second challenge is the differentiation between the primary and secondary renal lymphoma. Histopathology, which is the gold standard of the diagnosis,can differentiate between the renal lymphoma and RCC; however, it cannot make any differentiation between the PRL and SRL. This distinction between the primary and secondary renal lymphoma can only be reliably made by the strict application of the imaging based diagnostic criteria of the PRL(particularly, the absence of extrarenal visceral or nodal disease). Radiologists should be aware of the typical and atypical imaging features of the renal lymphoma, follow the proposed diagnostic criteria of the PRL and should advice imaging guided percutaneous biopsy for pathological confirmation which can avert superfluous nephrectomy. Early diagnosis and proper management is associated with good prognosis.

## Learning points

1. In disseminated lymphoma, the genitourinary system is the second most frequently affected system, and within the genitourinary system, the kidney is the most common affected organ.
2. Non-Hodgkin lymphoma, usually of the DLBCL subtype, is the most common secondary and primary renal lymphoma.
3. The imaging features of primary and secondary renal lymphoma are similar except that the SRL has associated widespread extrarenal disease. Absence of extrarenal visceral or nodal disease is one of the most important criteria in differentiating PRL and SRL.
4. Renal lymphoma has various radiological appearances, including multiple or solitary focal renal lesions, direct renal extension from the retroperitoneal lymphadenopathy, isolated perirenal disease and unilateral or bilateral diffuse renal infiltration causing nephromegaly without loss of the normal renal shape. The gold standard for diagnosis of renal lymphoma is renal biopsy.
5. PRL has to be distinguished from other common primary renal malignant lesions, such as renal cell carcinoma (RCC) because of the striking difference in their management.
